# Gels Obtained by Colloidal Self-Assembly of Amphiphilic Molecules

**DOI:** 10.3390/gels3030030

**Published:** 2017-08-03

**Authors:** Paula Malo de Molina, Michael Gradzielski

**Affiliations:** 1 Centro de Física de Materiales (CSIC, UPV/EHU) and Materials Physics Center MPC, Paseo Manuel de Lardizabal 5, E-20018 San Sebastián, Spain; 2 Institut für Physikalische & Theoretische Chemie—Stranski Laboratorium, Technische Universität Berlin, Straße des 17. Juni 124, 10623 Berlin, Germany

**Keywords:** gels, self-assembly, surfactants, amphiphilic polymers, rheology, colloids, micelles, microemulsions, vesicles

## Abstract

Gelation in water-based systems can be achieved in many different ways. This review focusses on ways that are based on self-assembly, i.e., a bottom-up approach. Self-assembly naturally requires amphiphilic molecules and accordingly the systems described here are based on surfactants and to some extent also on amphiphilic copolymers. In this review we are interested in cases of low and moderate concentrations of amphiphilic material employed to form hydrogels. Self-assembly allows for various approaches to achieve gelation. One of them is via increasing the effective volume fraction by encapsulating solvent, as in vesicles. Vesicles can be constructed in various morphologies and the different cases are discussed here. However, also the formation of very elongated worm-like micelles can lead to gelation, provided the structural relaxation times of these systems is long enough. Alternatively, one may employ amphiphilic copolymers of hydrophobically modified water soluble polymers that allow for network formation in solution by self-assembly due to having several hydrophobic modifications per polymer. Finally, one may combine such polymers with surfactant self-assemblies and thereby produce interconnected hybrid network systems with corresponding gel-like properties. As seen here there is a number of conceptually different approaches to achieve gelation by self-assembly and they may even become combined for further variation of the properties. These different approaches are described in this review to yield a comprehensive overview regarding the options for achieving gel formation by self-assembly.

## 1. Introduction

Self-assembly of amphiphilic molecules in solution can lead to a large variety of different colloidal structures [1], where these structures can have a profound effect on the macroscopic properties of these solutions. In this review we will, in particular, focus on systems which form gels by self-assembly of corresponding amphiphilic compounds. Of course, at very high concentrations all amphiphilic systems will form gel-type structures simply due to dense packing, where typically very stiff hexagonal, cubic or lamellar phases are formed [2,3]. However, we will explicitly not discuss such liquid crystalline phases of dense packing, but rather, focus in our review on more dilute systems in which basically the self-assembled structures lead to a gel-like behaviour of the systems. We will also exclude surfactant gels in which the surfactant is present in crystallized form. Especially for longer chain surfactants this can be achieved relatively easily and often leads to gelation, typically via formation of fibres [4] but also for the case of vesicular or lamellar structures, as for instance, known for the case of phospholipids (“gel phase”) [5]. Accordingly, our review is concerned with gels formed by reversible dynamic assemblies, where the properties depend largely on the molecular architecture of the amphiphilic molecules, which in turn, control the structure and dynamics of these self-assembled systems. 

Here it has to be noted that the definition of what is a gel is not necessarily undisputed and an authoritative source for a definition according to the International Union of Pure and Applied Chemistry (IUPAC) is that a gel is a “Non-fluid colloidal network or polymer network that is expanded throughout its whole volume by a fluid.” In addition, it is stated that “a gel has a finite, usually rather small, yield stress” [6]. However, the experimental confirmation of a yield stress is nothing straightforward [7], as practically that amounts to the situation of having a structural relaxation time τ_str_ longer than the experimental observation time window. 

For instance, the viscosity of self-assembled systems can become largely increased upon the formation of rodlike or wormlike micelles and once these are sufficiently long and/or concentrated viscoelastic surfactant solutions are formed [8,9]. Accordingly such systems can have zero-shear viscosities several orders of magnitude higher than the solvent itself, and this already at concentrations well below 1% [10]. However, such systems should be viscoelastic but not gels due to the expected finite structural relaxation time τ_str_. Their elastic properties are based on entanglements and for wormlike surfactant micelles, different to polymer networks, there is a finite reptation time. In addition, wormlike micelles are dynamic chains, that break with a characteristic time scale [11]. Nonetheless such systems (as well as fibres) have recently been discussed to have a gel-like collective response that arises from these topological interactions (entanglements) [12]. The crucial parameter here is the effective structural relaxation time τ_str_. Systems with an infinite (or at least substantially longer than the observation window) τ_str_ may be defined as self-assembled gels and they constitute interesting systems for formulations as they allow to exercise rheological control in a simple fashion. Accordingly systems with a finite but sufficiently long relaxation times (τ_str_ >> s) may for practical purposes be considered as gels, as in the relevant time and frequency range they respond similarly, which means mainly elastic and only to a much lesser extent viscous. 

In general, the shear modulus *G*_0_ and the zero-shear viscosity η_0_ are directly related to each other via the structural relaxation time τ_str_ via Equation (1).

(1) $$\eta_{0}=G_{0}\cdot \tau_{\mathrm{str}}$$

As stated before, dense packing of micelles [3] or vesicles [13] also leads to systems with pronounced rheological properties which typically have a yield stress, i.e., do not flow at all if not subjected to a minimum external stress. There are many principal ways of achieving gel-like behaviour by self-assembly and a larger number of them have been well established for surfactant assemblies [14]. Gel formation can also be achieved by appropriate surfactant mixtures and/or employing polymeric amphiphiles [15], or combinations thereof. Here in particular block copolymers of the Pluronic type (PEO-PPO-PEO; PEO: poly(ethylene oxide), PPO: poly(propylene oxide)) are frequently employed as gelating systems, which have the capacity to form gels already at rather low concentrations due to the fact that their large PEO head groups can bind a substantially larger volume of water than their PEO chains would have themselves. This enhanced effective hard sphere volume explains their facile gelation [16], where it has been noted that such gels disappear again upon the admixture of low Mw surfactants that dissolve the copolymer micelles [17]. In principle, they are just densely packed micelles, often in a liquid crystalline cubic arrangement, but the main practical difference to most conventional surfactants is the large amount of bound water, thereby facilitating gelation already at rather low surfactant concentrations (of 15–25 wt %). Furthermore, Pluronics are attractive systems from the point of applicability, as they have permission to be employed in almost any field of pharmaceutical or cosmetical applications [18]. 

So far we just focussed on the situation of gelation due to self-assembly. However, particularly interesting in that respect are naturally systems which are responsive to external parameters, such as pH, ionic strength, temperature, magnetic and electric fields, shear fields etc., as they allow to control the rheological properties externally and to construct smart systems that adapt correspondingly to such external stimuli. 

As seen from Equation (1) a main parameter is the shear modulus G_0_ that is directly related to the structural arrangement of the colloidal systems, as determined by the mesoscopic structure. From a simple network theory G_0_ is given by [19]:

(2) $$G_{0}=\nu \cdot {}^{1}N\cdot k\cdot T$$

where ^1^N is the number of cross-linking network points. In this theory it is simply assumed that each such network point can store one kT as elastic energy, in analogy with the energy stored per degree of freedom in an harmonic oscillator. ν is a parameter of the order one associated with a given specific structural arrangement. For an ideal network ν = 1 for an affine network and ν = (1 − 2/*f*) for phantom networks, where *f* is the cross-link functionality.

However, as stated above similarly important is the structural relaxation time τ_str_ that determines how long lived a given structural arrangement will be, which then is the key property that controls viscosity. Again, in a very simplified fashion this structural relaxation time can be approximated by:

(3) $$\tau_{\mathrm{str}}=A\cdot e^{E_{a}/k\cdot T}$$

where E_a_ is the activation energy required to break a cross-linking point, and A is the fastest possible break-up time (which is given by the inverse natural oscillation frequency of the network, for instance the movement of a hydrophobic sticker, and therefore typically is in the range of 10^−10^ s). E_a_ is the energetic effort for breaking up a given self-assembled connection, which in water is typically related to transferring a hydrophobic chain out of its environment in the hydrophobic assembly into the aqueous surrounding. This is known to be about 1.2 kT per CH_2_ group [20] and similarly values are also known for other hydrophobic moieties.

In the following, we will discuss in the various chapters different typical approaches to achieve colloidal gels by self-assembly of amphiphilic molecules, which are based on wormlike micelles, densely packed vesicles, self-assembling polymers, or bridging of surfactant structures by amphiphilic copolymers. 

## 2. Viscoelastic Networks of Wormlike Micelles

**Figure 1:**
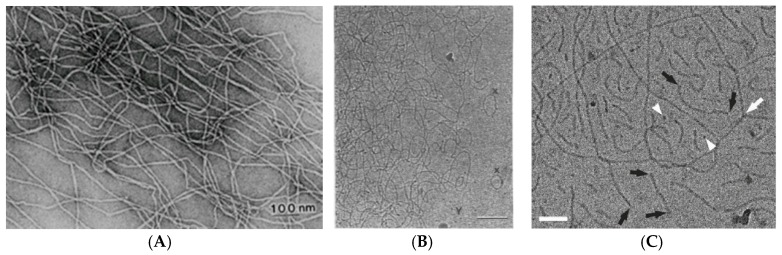
Electron micrograph of (**A**) a sample of 1 mM CTAB/1 mM NaSal [24] (With permission of Springer); (**B**) 50 mM CTAC/50 mM NaSal in 100 mM NaCl (scale bar: 100 nm) [27]; (**C**) NaOleate solution containing 15 wt % octyltrimethyl ammonium bromide (OTAB) (scale bar: 50 nm), white arrows indicate branching points and black arrows the end-caps [25].

Self-assembly of surfactants in form of micellar aggregates can lead to the formation of surfactant gels, which are an interesting class of molecular gels, without having to be of crystalline nature [21]. The formation of viscoelastic surfactant solutions may occur directly upon dissolution of a surfactant in aqueous solution, but is also often observed upon addition of an additive, e.g., of salt, hydrophobic counterions or cosurfactant to ionic surfactant solutions [22]. This empiric observation has been around for a long time and may already occur for surfactant concentrations well below 1 wt % [8]. Initially the structural origin of this interesting rheological behaviour was unclear but became clarified by intense research more than 30 years ago. It could be attributed to the formation of overlapping long wormlike micelles and was then also directly imaged by transmission electron microscopy (TEM) already more than 30 years ago for the cases of dimethyloleylamine oxide [23] or cetyltrimethylammonium bromide (CTAB)/sodium salicylate (NaSal) [24] (Figure 1A). Such systems for both in salt-free water but also in the presence of larger concentrations of salt, like shown in Figure 1B for the case of 100 mM NaCl. An interesting question here has been the branching of such long wormlike micelles but more recently it has been shown by cryo-TEM [25] (Figure 1C) that branching does occur in wormlike micelles and also has a profound effect on the rheological properties of these networks as the appearance of branching points increases the shear modulus G_0_ [26]. 

Viscoelastic and gel-like systems have also been intensely studied for mixtures of cationic surfactants, such as alkyltrimethylammonium or alkylpyridinium, with hydrophobic counterions such as benzoates, salicylates, or naphthoates. Similarly, anionic surfactants of the alkylcarboxylate or alkylsulfate type form very viscous solutions with counterions such as tetraalkylammonium salts [8,10]. However, it might be noted that often also the addition of simple salts to ionic surfactants can lead to a substantial enhancement of the viscoelastic properties of a given surfactant solution. This is simply due to the reduced head group size as the electrostatic screening increases. This then increases the packing parameter of the surfactant and thereby one has a shift to more elongated micelles (the packing parameter *p* is given as: *p* = v/(a_0_∙l_c_); where v is the volume of the hydrophobic part of the surfactant, a_0_ the head group area, and l_c_ a critical length which is roughly equal but less than the fully extended length of the hydrocarbon chain of the surfactant [28]). This effect is typically more pronounced for multi-valent counterions, as for instance demonstrated for sodium dodecyl trioxyethylene sulfate (SDTES) where the efficiency of the ions follows the rule: Al^3+^ > Mg^2+^ > Ca^2+^ > Na^+^ [29]. However, also the addition of simple NaCl to palmitylamido-sulfobetaine (PDAS) has been shown to help gelation properties where here in particular a thermoreversible gelation is observed which takes place upon heating from 30 to 40 °C and is linked to a transition of globular to wormlike micelles but only at very high surfactant concentrations of 1 mol/L [30]. 

The rheological behaviour of such systems of entangled wormlike micelles in oscillatory experiments can to a first order often be described by Maxwellian behaviour, as given by Equation (4) for the frequency dependence of the storage modulus G’ and the loss modulus G″, but at higher frequencies typically marked deviations are observed. These can be attributed to the fact that the wormlike micelles have a finite life time and, depending on the detailed molecular composition, will have a characteristic breaking time τ_break_ [31], which determines at which frequency one will observe deviations from the picture expected for simple wormlike objects (as they are present in polymer solutions).

(4a) $$G^{\prime }=G_{0}\cdot \frac{\omega^{2}\cdot {\tau_{\mathrm{str}}}^{2}}{1+\omega^{2}\cdot {\tau_{\mathrm{str}}}^{2}}$$

(4b) $$G^{\prime \prime }=G_{0}\cdot \frac{\omega \cdot \tau_{\mathrm{str}}}{1+\omega^{2}\cdot {\tau_{\mathrm{str}}}^{2}}$$

Typically, one observes that both, shear modulus G_0_ and zero-shear viscosity η_0_ follow a power law above a certain concentration c_0_:

(5a) $$G_{0}=A\cdot {\left(\frac{c-c_{0}}{c_{0}}\right)}^{\gamma }$$

(5b) $$\eta_{0}=B\cdot {\left(\frac{c-c_{0}}{c_{0}}\right)}^{\beta }$$

where c_0_ is an effective overlap concentration, A and B some system dependent pre-factors, and β and γ some system dependent power law exponents. γ depends mostly on the structural interconnection and typically is in the range of 1.5–3 while β may vary much more widely (between 1.5 to 8.5) as according to Equation (1) it also depends on the power law that applies to τ_str_. Typically β increases substantially with increasing electrostatic interaction of the micelles, being smallest for neutral systems and highest for unscreened charged systems [10].

As already discussed before normal solutions of wormlike micelles have a finite structural relaxation time τ_str_, which means they flow rather quickly under gravity. However, τ_str_ scales with the length of the hydrophobic chain of the surfactant, which normally is directly related to the kinetic exit time of the hydrophobic chain from the micelle and as the activation energy per CH_2_ unit is about 1.2 kT having 2 additional CH_2_ groups results in an increase by a factor ~10 (see Equation (3)). Accordingly, for long chain systems τ_str_ may move out of the experimental observation window. For instance one observes gel formation for erucyl dimethyl amidopropyl betaine (EDAB) for concentrations already above 10 mM and the shear modulus follows a power law of G_0_ ~ c(EDAB)^1.8^ for the surfactant concentration. Such a scaling is also in good agreement with theoretical predictions [32]. Of course, this behaviour is temperature dependent and upon heating to 60 °C one observes normal viscoelastic behaviour again [33], since τ_str_ depends strongly on temperature (scaling according to Equation (3)). This concept of having very long hydrophobic chains to enhance the elastic properties of wormlike micelles has also been extended to pseudo gemini surfactants composed of *N*-erucamido-*N*,*N*-dimethylamine (UC_22_AMPM) and maleic acid with a molar ratio of 2:1. This system was found to be quite temperature-sensitive and to be showing pronounced elastic properties already at concentration of 25 mM and, not surprisingly, these properties increase substantially upon increasing the surfactant concentration further. The most interesting aspect here is that it is quite sensitive to pH in the range of 6 and 7.5 [34], thereby indicating the importance of the charging conditions here.

In general, it is well known that gemini surfactants have a pronounced tendency for forming very elongated wormlike micelles [35]. As they have automatically two hydrophobic chains anchored within the wormlike micelle their structural relaxation times τ_str_ are much larger than those of the corresponding single chain surfactants. Accordingly, here one also produces easily viscoelastic surfactant solutions, where it has been found that the presence of a hydroxy group in the head group enhances viscosity and elastic properties, as seen in particular for the comparison of 2-hydroxyl-propanediyl-α,ω-*bis*-(dimethyldodecylammonium bromide) (12-3(OH)-12) and propanediyl-α,ω-bis(dimethyldodecylammonium bromide) (12-3-12) [36]. The same study also demonstrated the importance of the alkyl chain length as for 12-3(OH)-12 τ_str_ was in the range of s, while for 14-3(OH)-14 it moved into the range of hundreds or thousands of s, i.e., allowing for tube inversion and real gel formation (again this is not a surprising finding as one introduces 4 CH_2_ groups into the hydrophobic surfactant moiety and according to our above statements would expect to see an increase of τ_str_ by a factor ~100). 

In summary, it can then be stated that viscoelastic surfactant systems based on wormlike micelles allow for the formation of viscoelastic fluids, that in practice can mutate to gels. The main control parameter here is the structural relaxation time τ_str_, which in turn depends mainly on the length of the hydrophobic moiety.

## 3. Densely Packed Vesicle Gels

**Figure 2:**
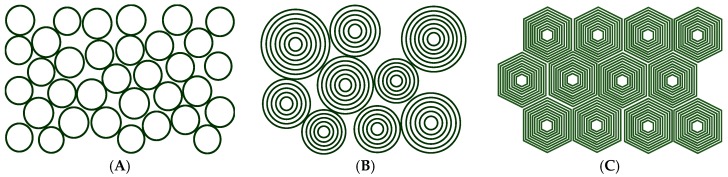
Sketch of different types of densely packed vesicle gels. (**A**) made up from unilamellar vesicles (ULVs); (**B**) made up from multilamellar vesicles (MLVs); (**C**) densely packed deformed vesicles at high concentration.

Another way of obtaining highly viscous or even gel-like systems is by having densely packed vesicles. This applies to unilamellar or multi-lamellar vesicles (ULVs, MLVs) that are closed surfactant bilayers (with one or many shells) (Figure 2). The rheology of vesicle systems has been reviewed some while ago [37], and they show typically enhanced viscosity compared to micellar systems and a shear thinning behaviour. Their enclosing of solvent allows to have densely packed systems of spherical objects, with the amphiphilic volume fraction typically in the range of 2–15 wt % of amphiphilic substance. In general, one may expect low volume fractions for ULVs and higher volume fractions with correlated layers in the MLVs. 

### 3.1. Vesicle Gels Based on Unilamellar Vesicles (ULVs)

**Figure 3:**
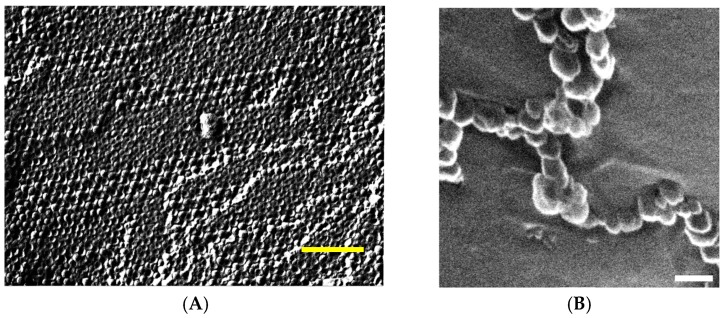
(**A**) Freeze-fracture transmission electron microscopy (FF-TEM) image of densely packed ULV in the system 182 Na isostearate/567 mM 1-octanol (the aqueous solution contained 20 wt % glycrol to facilitate the FF preparation (size bar: 200 nm) [41]; (**B**) cryo scanning electron microscopy (cryo-SEM) image of a C_18_–C_8_ gemini vesicle gel (size bar: 66.7 nm) [46].

We may first consider vesicle gels formed by ULVs. Such gels were already reported as early as 1968 by Fontell and Ekwall for densely packed vesicles observed in the system NaOleate/decanol/water [38]. However, it remained largely unnoticed, as the authors did not emphasize that interesting point in their study, which instead focussed on other aspects of surfactant self-assembly and phase behaviour. These investigations were taken up on basically similar systems in the 1990’s by Hoffmann et al. who not only studied the mesoscopic structure of these systems, but also their rheological properties in some detail [39,40]. These vesicle gels simply form spontaneously by diffusion of the cosurfactant into the oleate solution. Thereby the initial micellar oleate solution becomes first transformed into increasingly longer wormlike micelles with a corresponding marked increase of viscosity. This process occurs within the first 1–2 min and then is followed somewhat later by a transition from wormlike micelles into well-defined ULVs, where this process is typically completed after about 15–20 min and is accompanied by a gelation of the system, i.e., it possesses a yield stress then of ~200 Pa [41]. The shear modulus G_0_ is in the range of 1000–10,000 Pa, increasing with increasing surfactant concentration. It can be well explained via Equation (2), as now one has more vesicles since they become reduced in radius from 24 to 14 nm, in order to retain the packing volume fraction while having larger amounts of amphiphilic bilayer to disperse. The dense and highly ordered packing of vesicles can be seen well in the FF-TEM shown in Figure 3A. 

The observed structural progression can simply be explained by the change of the packing parameter p of the amphiphilic system that comes about by incorporating the octanol into the oleate system. The initially present globular micelles elongate into increasingly long rodlike micelles, which then finally transform to well-defined and rather monodisperse ULVs as followed and confirmed by time resolved small-angle neutron scattering (SANS) experiments [42]. The formed gel phase is isotropic and quite transparent and is found for oleate concentrations of ~150–400 mM and concentrations of 1-octanol of ~450–700 mM, which means that the amphiphilic film is largely composed of the cosurfactant 1-octanol and therefore also this bilayer is with ~2.2–2.5 nm rather thin. In addition, such vesicle gels can also be formed by adding other cosurfactants like heptanol, hexanol, or geraniol to an aqueous Na oleate solution, while shorter or longer alcohols lead to systems with much reduced elastic properties [40]. It might also be noted that here one is not restricted to oleate as surfactant, but the structurally related isostearate possesses a quite similar phase behaviour. It is interesting to note that subsequent NMR work on these well-defined ULV gels proved the existence of µm-size kind of “super-structure” or “grain-like structure” in these systems, which is several hundred times bigger than the individual vesicles, and which for instance for aspects of release of active agents from them should be of relevance [43]. A somewhat related investigation showed that NaOleate can also become transformed into a gel phase by addition of *N*,*N*-*bis*(carboxylatomethyl) glutamate (GLDA). However, in that case not the formation of ULVs is responsible for gelation but instead long and stiff fibrils of lamellae are at its origin [44]. 

Further work also showed that the structural features of the vesicle gel can be retained during silication, where the initial vesicle gel contained in addition tetraethyl orthosilicate (TEOS) as a silica source. The TEOS then hydrolyses more slowly than required for the vesicle gel formation to take place, therefore not interfering with it [45]. Interestingly the incorporation of the silica network leads to a reduction of the elastic properties, which becomes very pronounced beyond a certain critical TEOS concentration [45]. 

Some time ago, the formation of vesicle gels of strings of vesicles was reported for the case of gemini surfactants, where it was speculated that this percolating system of vesicles comes about by the protrusion of small chains from the vesicle surface (Figure 3B) [46]. This then renders the vesicles attractive to each other and the bridging of two vesicles by the gemini surfactant thus leads to the formation of strings that for high enough concentration yields a space-filling percolated network. Such behaviour was observed for a number of gemini surfactants, all having in common a large asymmetry with respect to the length of their two hydrocarbon chains and gel strength and yield stress were found to depend markedly on the molecular structure of the gemini surfactant. 

When getting more and more densely packed, one may expect that the charged vesicles escape from this crowded situation by deflation and formation of bi- or multilamellar vesicles. This mechanism actually has been described recently by theory and supported by experimental evidence [47]. 

### 3.2. Vesicle Gels Based on Multilamellar Vesicles (MLVs)

**Figure 4:**
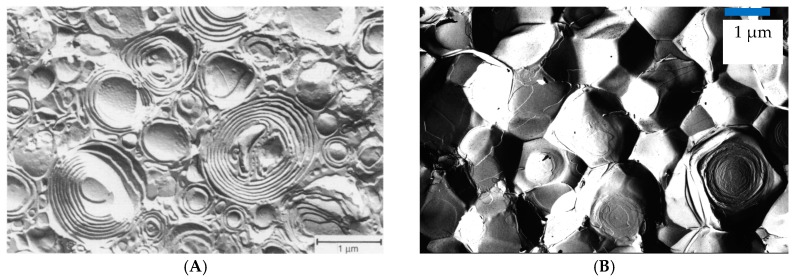
FF-TEM micrographs of the systems: (**A**) 90 mM TDMAO/10 mM TTABr/220 mM 1-hexanol (Reproduced (“Adapted” or “in part”) from [49] with permission of The Royal Society of Chemistry.); (**B**) 360 mM TDMAO/40 mM TTABr/780 mM 1-hexanol/700 mM NaCl.

Of course, the concept of densely packed spherical colloids just described for ULVs can be extended to multilamellar vesicles (MLVs) and actually here the formation of such gellike and viscoelastic systems has been reported much more often. Such vesicle gels cannot only be formed by spherically shaped ULVs and MLVs (Figure 4A) but for higher concentrations of amphiphilic material the vesicles have to deviate from a spherical shape in order to allow for a more dense packing (see Figure 2C and Figure 4). Such “deformed MLVs” then are of polyhedral shape, which allows for the correspondingly required more dense packing. Examples for such structures are depicted in Figure 4B. If made from phospholipid such liposome gels of MLVs are also of high interest for practical formulations in the context of delivery systems [48]. 

An example for such densely packed MLV is given for the zwitterionic surfactant tetradecyl-dimethyl amine oxide (TDMAO) that by addition of a cosurfactant like hexanol or heptanol becomes transformed into a state of vesicles or lamellae. By protonation or substitution of the TDMAO by the cationic tetradecyltrimethyl ammonium bromide (TTAB) this system can be shifted further into the state of MLVs [50]. Already at a surfactant concentration of 100 mM (~2.5 wt %) the formation of MLVs results in pronounced elastic properties with a shear modulus G_0_ in the range of 10–100 Pa and the formed systems even exhibit a yield stress. This is due to the fact that one has here µm sized onion-type MLVs that are densely packed, as seen in Figure 4A [49]. An interesting observation is that G_0_ is very sensitive to the charging of the systems. While the uncharged system shows basically no gel-like behaviour, already the presence of 1 mol % charged surfactant leads to viscoelastic properties and raising this value to 4 mol % leads to a substantial increase of G_0_ to ~40 Pa, while further charging then has no effect and G_0_ remains constant thereafter. It is also interesting to note that the rheological behaviour is almost the same whether one charges the system by protonation or by substituting TDMAO by TTAB, indicating that it is a purely electrostatic effect. Of course, as the rheological properties here depend so strongly on electrostatics the addition of salt then leads to a substantial reduction of the viscoelastic properties again. Upon increasing the surfactant concentration by a factor 4 and having a rather high salinity of 700 mM NaCl one observes the formation of densely packed multifacetted vesicles as depicted in Figure 4B (the high salinity here reduces the electrostatic repulsion between the bilayers and thereby facilitates their dense packing). 

**Figure 5:**
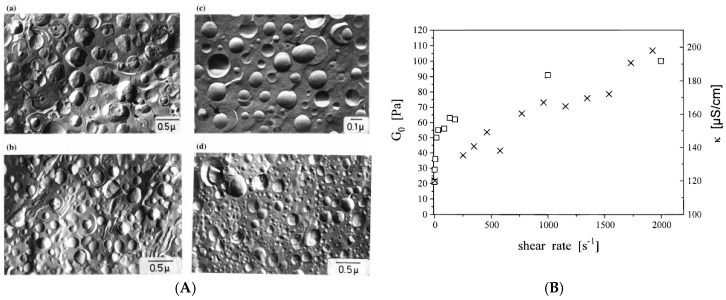
(**A**): FF-TEM micrographs of the system 90 mM TDMAO/10 mM TTABr/220 mM 1-hexanol: (**a**) immediately after shearing the sample for 1.5 h at a shear rate of 200 s^−1^; (**b**) 2000 s^−1^; (**c**) 4000 s^−1^; (**d**) after allowing the system depicted in (**c**) to relax under stirring for 12 days; (**B**): Shear modulus G_0_ (□) and electric conductivity during shear in vorticity direction (×) versus shear rate of the pre-shear for the same system. The vesicle solution was sheared at the given shear rate until the apparent shear viscosity indicated a steady state. Then, shearing was stopped and the modulus was measured in an oscillation experiment (Original in [51]).

It might be noted that this MLV TDMAO/TTAB/1-hexanol system can elegantly be changed in its structure by application of shear forces, where with increasing shear rate one reduces the number of lamellae in the MLVs until at very high shear rates of several 1000 s^−1^ finally unilamellar vesicles are present (Figure 5A). Of course, at the same time the number of vesicles increases substantially and this also leads to an increase of the shear modulus G_0_ (Figure 5B) which is in agreement with Equation (2) and demonstrates that here the number of effective network points has increased [51]. It might be noted that this transformation of vesicle morphology is basically irreversible (i.e., no relaxation process to any of the other structures was observed), but therefore it also remains unclear which state here is really thermodynamically preferred. Of course, it should be noted that size control of MLVs by shear had already reported before [52] but not in the context of parallel rheological control. 

A somewhat related system is also based on TDMAO but uses the fact that TDMAO is a weak base, which can become protonated by a strong acid. This was accomplished by mixing it with perfluorolauric acid (PFLA) and this catanionic surfactant system forms birefringent gels for surfactant concentrations higher than 50 mM and a molar content of PFLA of 80–90%. 100 mM systems have shear moduli of ~1000 Pa and the structural investigations show the presence of MLVs here, but ones that are in the crystalline state at room temperature and only melt around 50 °C, as also the pure PFLA melts at 55 °C [53]. 

Another interesting observation on the TDMAO/cosurfactant system was that one can also induce the formation of a densely packed MLV gel by the addition of a hydrocarbon (here decane) to a concentrated solution of TDMAO and benzyl alcohol, where the structure and rheological properties are controlled by the amount of decane contained [54].

As indicated the formation of MLVs is often linked to the presence of a cosurfactant. Accordingly, similar systems have also been described for the case of the classical surfactant SDS and cetyl alcohol. The reason for the gellike behaviour here could be attributed to jammed packing of uni- or multi-lamellar vesicles as determined mainly a combination of ^1^H and ^13^C-NMR [55]. For phospholipids such gels are formed from lecithin upon the addition of sodium deoxycholate, sodium cholate, sodium taurodeoxycholate, or sodium taurocholate, where the rheological properties depend on the precise ratio of bile salt and lecithin. Robust gels are formed already around molar ratios for bile salt/lecithin of ~0.2 and lecithin concentration of 400 mM [56]. 

**Figure 6:**
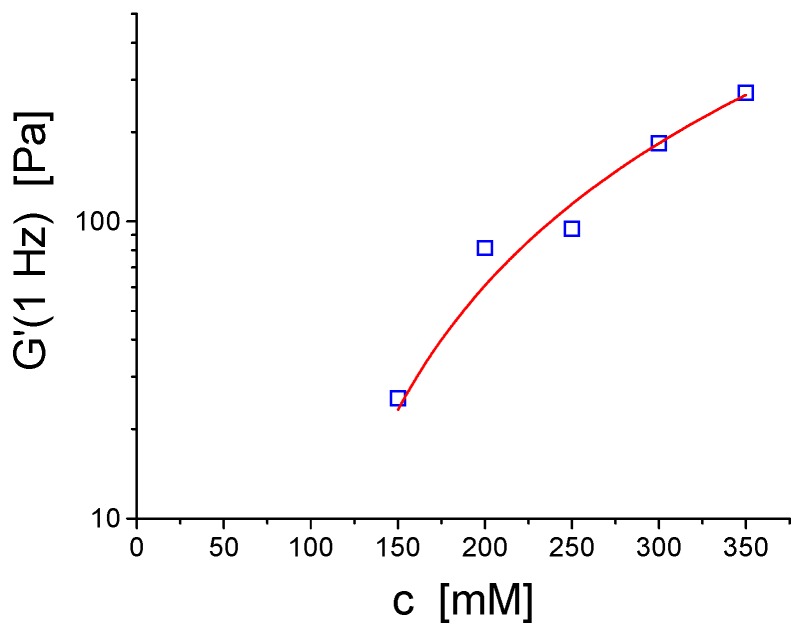
Storage modulus G′ as a function of total concentration for vesicles gels composed of Brij30 (technical grade C_12_E_4_) and 4 mol % DTAB, solid line: G′(1 Hz) = A × (c − c_0_)^x^; c_0_ = 76 mM, x = 1.87.

In another type of system based on a classical nonionic surfactant C_12_E_4_, that is well known to form vesicles [57] gelation was induced and controlled by the addition of cationic dodecyltrimethyl ammonium bromide DTAB. Yield stress and elastic modulus increase with increasing content of DTAB, where a maximum is already achieved around 4–5 mol % substitution. Of course, the elastic properties can further be controlled by the total concentration of surfactant as shown in Figure 6 [58]. A similar phase and rheological behaviour was observed for another nonionic surfactant of somewhat longer chain length and in addition containing ethylhexylglyceride as cosurfactant. Here the addition of SDS resulted in the formation of a vesicle gel with a yield stress, which could be explained by a simple electrostatic model for the bending constant of the bilayers [59]. Later work showed a similar behaviour upon admixing the anionic surfactant sodium *bis*(2-ethyl hexyl)sulfosuccinate (AOT) [60]. 

Furthermore, it could also be shown that charging of the C_12_E_4_ bilayers by means of adding anionic perfluorolauric acid (PFLA) leads to the formation of vesicle gels for concentrations around 10 wt %, where yield stress and elastic modulus increase substantially with increasing content of ionic surfactant PFLA [61]. In a related later study the charging of the C_12_E_4_ system was done by adding the amphiphilic anionic dye sodium 4-phenylazobenzoic acid (AzoNa). The obtained vesicle gels were responsive to temperature, pH and light, increasing in elastic properties with increasing temperature in a reversible way and being stable in the pH range of 7 to 11, while losing their gel properties outside this pH range [62]. Illumination by UV light initiates a transition from trans to cis conformation of the AzoNa which promotes bilayer formation thereby leading to gelation of the system. This process then can be switched back by illuminating with visible light [62], i.e., this is a light-responsive self-assembled gel. It might be added that a similar light responsive formation of a hydrogel could also be achieved for the case of the cationic surfactant, alkyltrimethylammonium bromide (C*_n_*TAB, *n* = 12, 14, 16, and 18) via the addition of sodium azobzenzene 4,4′-dicarboxylic acid (AzoNa_2_), which can be switched from cis to trans conformation by UV illumination. However, the strong gels formed there are not due to vesicle formation but the reason is the formation of very long (many µm) fibers in the presence of the cis-AzoNa_2_ [63]. 

A classical way of forming vesicles is the mixture of cationic and anionic surfactants, i.e., for catanionic surfactants [64]. By a variation on that theme it has been shown that the mixture of tetradecyl or dodecyl trimethyl ammonium hydroxide (TTAOH/DTAOH) with the 2-hydroxy-1-carboxy-naphthoate (HCN). This leads to salt free systems where HCN constitutes the hydrophobic counterion. For both surfactants (DTA and TTA) one observes already at 100 mM concentration around equimolar mixing or some HCN excess the formation of MLVs which form a weak gel with about 20 Pa storage modulus and exhibiting a yield stress [65]. A similar behaviour had been observed before when employing CTAOH as cationic surfactant and here it was interesting to note that for lower amounts of admixed HCN a viscoelastic phase of wormlike micelles is formed, where this viscoelastic solutions show a very similar storage modulus at high frequency as the MLV gels, but not having their yield stress [66]. In that context, it is also interesting to note that these pronounced elastic properties of a typical gel system are only observed for the salt-free C*_n_*TA/HCN systems, while mixing their salts (which produces the equimolar amount of salt, e.g., NaBr) leads to a reduction of the elastic modulus by a factor 100 while at the same time one observes the formation of MLV but together with other locally lamellar structures [67]. Apparently the elastic properties of this system are largely controlled by the electrostatic interaction. 

It might be noted that catanionic surfactants are most known for their spontaneous formation of well-defined ULVs but at higher concentration will often form MLVs, which then are in dense packing forming gels, as for instance described for the case of CTAT/SDBS [68].

## 4. Hydrophobically Modified Polymers

**Figure 7:**
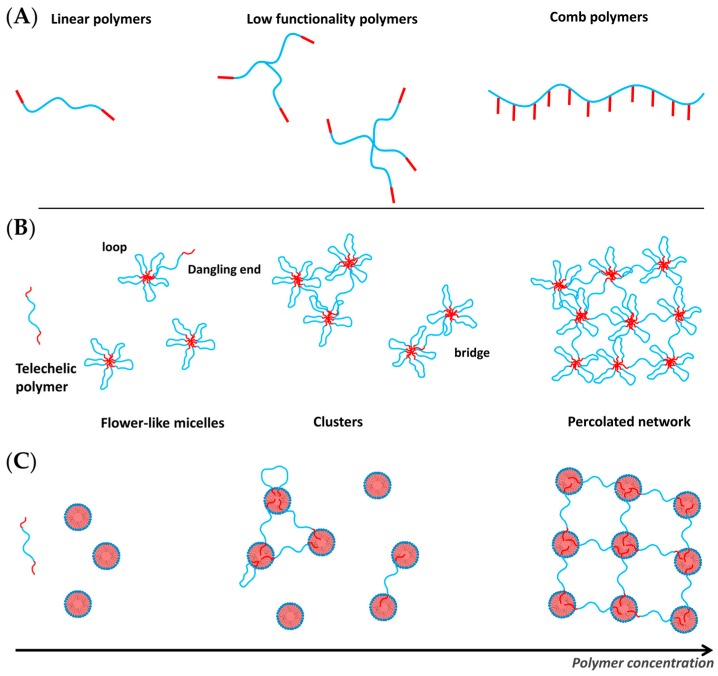
Schematic representation of (**A**) the polymer architecture of linear and low functionality telechelic polymers and comb-type hydrophobically modified polymers; association of telechelic polymers in (**B**) aqueous solutions and (**C**) with microemulsions as a function of the polymer concentration.

So far we have considered gels that are formed by surfactant self-assembly due to the aggregate structure, i.e., long worm-like micelles and densely packed micelles. However, in general, one may achieve similar self-assembled systems by employing amphiphilic copolymers. A particularly interesting class of polymers in that context are hydrophobically modified water-soluble polymers (see Figure 7). Here the hydrophobic modification is typically an alkyl chain of similar length as in surfactants and, therefore, has a tendency to associate with other such chains. Depending on the number of hydrophobic modifications along the polymer backbone, their concentration and the flexibility of the polymer, these hydrophobic chains may self-assemble like a micelle or yield connecting hydrophobic contact points. The structure and rheological properties differ significantly for the different polymer architectures. Hydrophobically modified water-soluble polymers can be classified into three main groups (Figure 7A):

1. Telechelic polymers, which are linear polymers end-capped with two stickers, alkyl chains or short hydrophobic blocks;
2. Low functionality multisticker polymers;
3. Multisticker grafted polymer chains with randomly distributed pendant hydrophobes along the hydrophilic chain (comb polymers).

Of course, beyond the architecture here it is also interesting to have systems where this hydrophilic/hydrophobic balance depends on external parameters, such as temperature, pressure, pH, ionic strength, etc., i.e., stimuli responsive systems. 

### 4.1. Telechelic Polymers

The self-assembly and mechanical properties of telechelic polymers, or ABA triblock copolymers (with A being hydrophobic and B being hydrophilic), is relatively well understood. The hydrophilic block most widely used is poly(ethylene oxide) [69,70,71,72] but there are examples with other chemistries, such as poly *N,N*′-dimethylacrylamide [73,74]. The hydrophobic end-group is typically a hydrocarbon alkyl chain [72,74,75]. However, also fluorocarbon alkyl chains [76] and hydrophobic polymer blocks (for example, polystyrene [77] and polybutadiene [78]) have been explored. Of course, one may also have a similar situation for ABC copolymers with A and C being two different hydrophobic units, which has also been discussed and studied in some detail [78]. The current understanding of the self-assembly and mechanical properties of telechelic polymers has been collected in a recent review by Chassenieux et al. [79]. Telechelic polymers aggregate at low concentrations into flower-like micelles [70], where different techniques yield different micellar aggregation numbers for a given system. For instance, fluorescence gives lower numbers than those obtained by static light or neutron scattering, and these in turn are slightly lower than the ones from dynamic light scattering (DLS) and viscosimetry [72,80,81]. However, they all agree that, compared to surfactant micelles, associative polymer micelles have lower aggregation numbers ($N_{agg}<50$). Theoretically, the aggregation number of flower-like micelles results from balancing the interfacial energy, configurational entropy, and excluded volume interactions in the corona against the deformation energy of the hydrophobic chains in the core. The polydispersity of the micelles arises from thermal fluctuations [82]. For long hydrophilic chains, the loop formation does not affect significantly the free energy and micelles of telechelic chains theoretically are predicted to have the same aggregation number as a solution of double the number of chains with one hydrophobic end-cap group [69]. This result has been in fact found experimentally by Sérero et al. [70].

At higher polymer concentrations, as the number density of micelles increases, they come closer together and polymer chains are able to reversibly form bridges between micelles leading to the formation of clusters [83] (Figure 7B). The cluster size grows with increasing concentration until the percolation concentration, where one cluster spans the entire volume and a transient network is formed. The rheological properties of these networks have been studied in detail [71,84,85,86]. Linear oscillatory shear measurements exhibit viscoelastic behaviour with one relaxation time and a high frequency modulus that can be described by a Maxwell model (Equation (4)) with one single relaxation time $\tau_{str}$ and a plateau elastic modulus $G_{0}$ (Equation (2)). The structural relaxation time $\tau_{str}$ is related to the residence time of the hydrophobic sticker in the micelle. Thus, the experimental structural times are very similar to the relaxation times determined for micellar kinetics for surfactants with the same chain length and increases strongly with increasing chain length of hydrophobic sticker [85]. Variation of the end-group chemistry consequently affects the sticker residence time in the micelles, i.e., hydrophobically modified polymers with fluorocarbon chains have longer lifetimes of the bridges than polymers end-capped with alkyl chains of the same length [76]. The elastic modulus in case of flexible and unentangled chains is expected to depend on the number of bridges. In the simplest case of rubber theory each bridge contributes 1 kT to the elasticity (see Equation (2)). Thus, predicting the value of $G_{0}$ depends on the ability to estimate the fraction of bridges versus loops and dangling ends [87]. In terms of the non-linear rheology, they often exhibit shear thickening behaviour prior to a sharp decrease in viscosity of several orders of magnitude [71,88,89].

**Figure 8:**
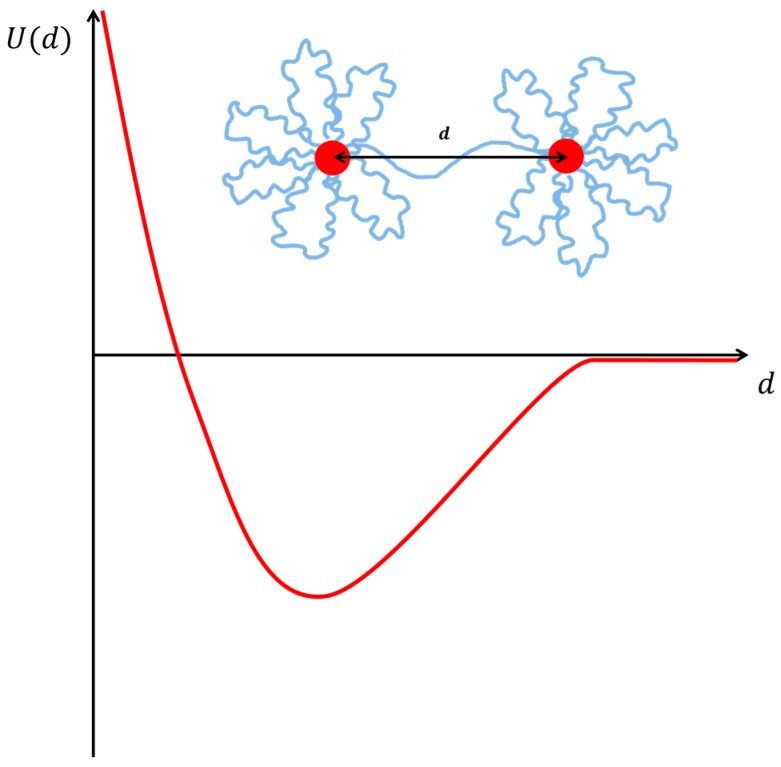
Schematic representation of the interaction potential between micelles (microemulsions) that are decorated and bridged by a telechelic polymer. The interaction has an effective attractive interaction between the micelles, due to the bridging, and a repulsive interaction, due to the steric repulsion between the micelles induced by the presence of the water soluble polymer that decorates the micelles.

The structure of flower-like micelles fits well to a model for star polymers [90]. With this model one can well describe the small angle scattering data of isolated micelles at low concentrations. In the concentrated regime micelles are experimentally found to interact repulsively [70,74,91], due to the excluded volume of the bridging chains. However, theoretically an attractive component due to bridging is expected [92] (Figure 8). The strength of the attractive interaction has been predicted to be about 1 $k_{B}T$ per chain, regardless of the lifetime of the bridges. At constant concentration, an increase in the strength of the attraction leads to phase separation [92].

### 4.2. Amphiphilic Polymers with Multiple Hydrophobic Stickers

Polymers with many hydrophobic groups attached to a hydrophilic backbone associate in more complicated structures since the hydrophobes may associate with other hydrophobes of the same polymer molecule or of different molecules. Depending on the polymer architecture and concentration, these polymers also form transient networks. They show a strong viscosity increase in the semi-dilute regime that is more pronounced if the grafting density is higher or the length of the stickers is increased. These solutions exhibit viscoelastic behavior. Their rheological response presents a broader distribution of relaxation times compared to the HM end-capped linear polymers [73]. A theoretical work proposed by Rubinstein and Semenov [93] predicted that the dynamics in the dilute regime is mainly controlled by intramolecular association. With increasing concentration, the polymer chain dynamics can be described by a sticky Rouse model for unentangled polymers and a sticky reptation model for entangled polymers. Experimental data of HM neutral polymers, such as polyacrylamide [94] or poly(*N,N*′-dimethylacrylamide) [73], agree with the theoretical predictions. 

In the case of just a low number of stickers per polymer, i.e., more than 2 but less than 10, considerably less work can been found in the literature. Associative star block copolymers—such as poly(acrylic acid)-block-polystyrene (PAA-*b*-PS)_4_ [77], poly(ethylene glycol)-*b*-poly(*N*-isoprylacryl-amide) (PEO-*b*-PNiPAAM)*x*, with *x* = 2–8 [95] or (PEG-*b*-PLLA) [96,97]—aggregate in aqueous solutions und undergo a sol-gel transition at a critical concentration in the same way bifunctional polymers do. In general, polymers with higher number of associative groups form networks more effectively due to lower intramolecular association. However, more systematic studies are needed in order to understand the effect of the polymer architecture on the network properties. Recent investigations on 3-arm and 4-arm end-capped polymers showed that the polymer functionality impacts substantially the rheological properties of the network in terms of the network elasticity. Higher functionality leads to higher connectivity and thus to higher plateau moduli. The viscoelastic behaviour is still almost of Maxwell type with one relaxation time given by the sticker length [74].

The interactions of associative groups that give rise to the transient junctions can be other than hydrophobic interactions. Sophisticated end-functionalization of polymers has led to a library of associating polymers that bond through noncovalent physical interactions such as metal−ligand coordination [98,99,100], hydrogen bonding, [101] and host−guest interactions [102]. Compared to the classical telechelic polymers, the multiplicity of the network links is given by the type of physical bonding and it is generally less than 5.

Also, although most of the work has been done on water based systems, polymers that associate in non-polar solvents havebeen studied. They form micelles and networks with the same governing physics as in the water-based systems. For instance, triblock copolymers with a middle block soluble in paraffin oil and insoluble polystyrene end-blocks are able to associate into interconnected micelles in paraffin oil [103,104]. Analogously, block copolymers with an oil soluble middle block and water soluble end-groups are able to bridge and network reverse swollen micelles in oil [105,106] in the same way that occurs in the water based systems described below. 

### 4.3. Stimuli Responsive Copolymers

All the aspects discussed above apply similarly to systems where the hydrophilicity of one block becomes switched on or off by an external parameter, such as temperature, pressure, pH, or ionic strength—and, of course, such switchable systems are very interesting. Accordingly, here many concepts have been presented and in our review we want to focus purely on such where by the change of an external parameter the hydrophilicity/hydrophobicity can be switched and thereby the rheological properties of the systems. As an example for the case of temperature sensitive systems this means that one of the blocks has to possess a lower (LCST) or an upper (UCST) critical solution temperature [107]. As the field of stimuli responsive polymers and their effect on macroscopic properties is a very wide one we want in the following only discuss some exemplary cases relevant for gelation, for instance arising from interconnection of self-assembled entities. 

Most frequently ABA triblock copolymers have been employed where the block A is the switchable block. A good example for such a system are copolymers with A = 2-(diisopropyl-amino)ethyl methacrylate), DPA or 2-(diethylamino)ethyl methacrylate), DEA; B = 2-metha-cryloyloxyethyl phosphorylcholine, MPC, where DEA and DPA possess a LCST, which means that they become hydrophobic above a certain temperature and then form hydrophobic domains (see Figure 7B). This means that above this temperature and for concentrations about 10 wt % gelation takes place. These gels then dissolve again at lower pH due to protonation of the A block [108], i.e., are in addition pH-sensitive. A slight variation of this system then was done with PNIPAm as hydrophobic A block which forms gel-like systems at temperatures above 35 °C [109]. An interesting extension of this work then lead to thermogelling systems that contain PNIPAm as LCST A block. In addition, these polymers contained a S–S bond in the center of the copolymer, which then allows for chemical disintegration of the gel by a reduction reaction, that for instance can be done under very mild conditions by the tripeptide glutathione [110]. A similar redox-responsive system has been constructed for the case of NIPAM-*b*-PDMA-*b*-PNIPAM or PDEGA-*b*-PDMA-*b*-PDEGA copolymers, which were obtained by the RAFT procedure and correspondingly contain a central trithiocarbonate unit. The initially formed gel can be broken by aminolysis and the formed thiol capped copolymer micelles can be cross-linked reversibly by oxidation [111]. Of course, the gelation concentration depends on the detailed molecular architecture of the ABA copolymer and for PLGA-PEO-PLGA was shown to be mainly dependent on the block lengths [112]. 

Of course, in addition to just having temperature responsiveness one may also switch hydrophobicity by pH. This was for instance demonstrated for the case of (PDEAEM_25_-*b*-PEO_100_-*b*-PPO_65_-*b*-PEO_100_-*b*-PDEAEM_25_) pentablock copolymers. By SANS experiments it could be shown that above the LCST at 70 °C one has at pH 7.4 micellar aggregates that form a cross-linked gel upon raising the pH to 10.5 [113]. However, the pH-dependence can also arise from the center block, as demonstrated for the case of PMMA-PDMAEMA-PMMA, where above a certain polymer concentration gels are formed in aqueous solution due to the formation of bridged hydrophobic domains. However, this mechanism is only well working at intermediate pH of ~4, where the PDMAEMA chain is almost fully charged and thereby fully stretched. At higher pH the PDMAEMA chain becomes neutralized and therefore is no longer able to bridge and at lower pH one has automatically a substantial increase of the ionic strength which then screens the electrostatic repulsion within the PDMAEMA chain and therefore much reduced chain elongation. Accordingly, one can switch by pH from a gel state at pH ~ 4 to a sol state at higher or lower pH [114].

A coupled pH/temperature responsiveness of a sol-gel transition has been observed for linear triblock copolymer, poly(methoxydi(ethyleneglycol) methacrylate-*co*-methacrylic acid)-*b*-PEO-*b*-poly(methoxydi(ethylene glycol) methacrylate-*co*-methacrylic acid) (P(DEGMMA-*co*-MAA)-*b*-PEO-*b*-P(DEGMMA-*co*-MAA)), and by appropriately changing pH and temperature one can obtain successive sol-gel and gel-sol transitions in a narrow pH range [115]. However, here exist also other approaches for introducing thermoresponsiveness and for instance it can also be obtained by having hydrophobic dipeptides (dityrosine end groups) that end-cap a PEG chain. The dipeptides then form β-sheet fibrils that lead to a gel-sol transition near body-temperature [116].

Of course, there are many more ways to employ amphiphilic polymers for the formation of polymeric hydrogels and we have depicted here only some, which are more directly related to our general theme of surfactant based hydrogels. However, the interested reader may here be referred to recent reviews that focus on this topic of polymeric hydrogels [117,118]. 

## 5. Micellar Systems, Microemulsions or Vesicles Cross-linked by Amphiphilic Polymers

As seen in the chapter before amphiphilic copolymers can self-assemble into network gels by themselves, but such self-assembly can substantially be altered and strengthened by the presence of surfactant. This means that one may cross-link micelles or microemulsion droplets (which for simplicity one may consider as micelles swollen with a hydrophobic compound) by the amphiphilic copolymer and similarly vesicles may become cross-linked by such polymers. It might be mentioned that our review here is not complete with respect to the options existing for using mixtures of surfactant and polymer to achieve gel type systems, as that can also be achieved by mixtures of polyelectrolyes and surfactants as they have been reviewed recently [119,120]. A well-established case for such a system is hydroxyethyl cellulose (HEC), which can be cationically and hydrophobicially modified and by combining with oppositely charged anionic surfactants one can form highly viscous systems, which may exhibit gel-like behaviour already at very low concentrations (whereas the pure HEC and surfactant solutions are water viscous). At equimolar mixing of charges one may observe precipitation but upon approaching this two-phase region a very marked increase of viscosity by several orders of magnitude will take place [121], typically in a rather narrow concentration range. The formation of gels strongly depends on the constitution of the polymer and its concentration. Having oppositely charged groups present and also the presence of hydrophobic modification on the polymer reduces the concentration at which gelation is observed [122]. Without the electrostatic interaction no more pronounced interaction is observed and accordingly no gelation takes place. 

### 5.1. Surfactant Micelles Interacting with Amphiphilic Polymers

A rather classical case of viscosity enhancement or gelation is observed in mixtures of surfactants with hydrophobically modified polymers, typically a water soluble polymer where alkyl chains are present as hydrophobic side-arms. 

Non-ionic and ionic surfactants have a strong affinity for the hydrophobic domains formed by associative polymers. The zero shear viscosity as a function of the surfactant concentration has a maximum at a concentration close to the cmc of the pure surfactant solution. Assuming that the surfactant has no interaction with the hydrophilic chain, the addition of surfactant to a solution of HM-polymer of constant concentration results in the creation of mixed micelles. Their rheological properties arise mainly from two effects: (1) the lifetime of the stickers in the mixed micelles and (2) the number density of mixed micelles (cross-linking points) and hence the distance between them.

For aqueous solutions of telechelic polymers that are, as explained above, quasi-Maxwellian fluids, the changes on the network relaxation and the elastic modulus are easy to observe [123]. Higher surfactant concentrations increase the number density of the micelles in the system, which brings the micelles closer together, thereby enabling the polymer to form bridges. At the same time, there is a lower probability of having many stickers in the same micelle. The net effect of both opposing factors is a peak in the modulus as a function of surfactant concentration. At high polymer concentrations, where loops are less probable, the addition of surfactant has a rather limited influence on the formation of bridges, resulting in an almost immediate drop of the modulus without appreciable prior increase [123]. 

Now let’s consider the other case, where telechelic polymer is added to a surfactant solution of constant concentration with already formed micelles. In this case, the addition of polymer leads to the decoration followed by the subsequent interconnection of micelles with the corresponding increase in viscosity and elastic modulus. In terms of the structure, subsequent addition of telechelic polymer to a micellar solution doesn’t significantly change its structure but induces repulsive interactions between them [124], as well as attractive ones due to the capacity for bridging micelles. A typical curve for the potential energy between two micelles (or similarly microemulsion droplets) in the presence of telechelic polymer is given in Figure 8. 

**Figure 9:**
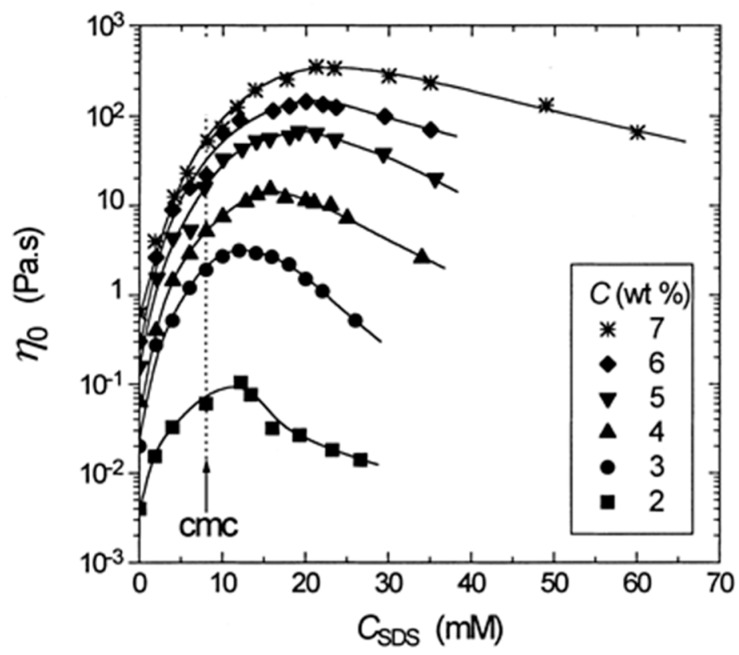
Effect of sodium dodecyl sulfate (SDS) concentration on the zero-shear viscosity of aqueous hydrophobically modified polyacrylamide (HMPAM) solutions of different concentration C [126].

In the case of HM-grafted polymers, the viscosity and the relaxation time undergo a maximum as a function of the added surfactant concentration. Compared to the case of regular telechelic polymers, the increase of the number density of micelles results in fewer cross-links and the corresponding decrease in the elasticity. Therefore, the non-monotonic variation of the viscosity arises from variations in the residence time in mixed micelles and will vary depending on the nature of the surfactant—it’s length and head group. The concentration at the viscosity maximum correlates with the CMC of the surfactant [125,126] and increases substantially with increasing concentration of the hydrophobically modified polymer, as shown in Figure 9 for the case of hydrophobically modified polyacrylamide (HMPAM) with sodium dodecyl sulfate (SDS).

However, micelles may have other shapes than spherical. The addition of telechelic polymers to worm-like micelles also leads to additional interconnection between the micelles. In this case, worm like micelles already have viscoelastic behaviour arising from the micelle´s interconnection. The addition of telechelic polymer results in a viscoelastic fluid with two coupled networks characterized by two relaxation times, one related to the worm-like micelle network and one related to the telechelic polymer bridging different micelles [127]. In such systems one observes two characteristic rheological relaxation times with two corresponding elastic moduli—to be described by two Maxwell fluids. The fast mode can be associated to the network of telechelic active chains that bridge two micelles, while the slow mode arises from the network of entangled wormlike micelles [128]. It has been demonstrated by SANS that, in a similar way as for spherical micelles, the telechelic polymer induces both an effective attractive interaction between the micelles, due to the bridging of the micelles, and a repulsive interaction, due to the steric repulsion between the micelles induced by the presence of the water soluble polymer that decorates the micelles [129]. 

### 5.2. Microemulsions Interacting with Polymers

**Figure 10:**
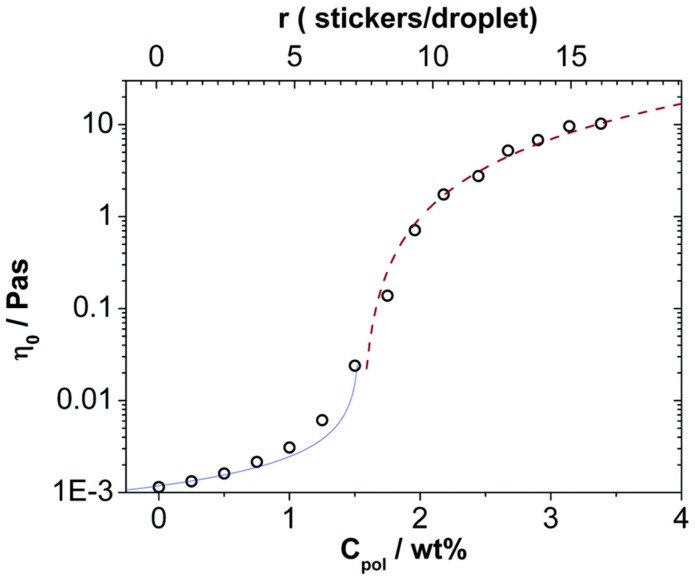
Zero-shear viscosity η_0_ at 25 °C of the mixtures of a microemulsion (100 mM TDMAO/35 mM decane in water) as a function of the concentration of C_18_-EO_150_-C_18_ measured with a capillary viscometer until a concentration of 2 wt % and with the instrument AR-G2 above this concentration. Solid line: η_0_ = 0.0016((1.54 − c)/wt %)^−0.7^. Dashed line: η_0_ = 3.6((c − 1.54)/wt %)^1.7^ ([136]—Published by The Royal Society of Chemistry).

Microemulsions (ME) are homogeneous, thermodynamically stable and finely dispersed mixtures of oil and water stabilized by a surfactant film [130]. MEs may occur in the form of oil-in-water (O/W) and water-in-oil (W/O) droplets, or as bicontinuous structures [131]. O/W microemulsions are attractive formulations for encapsulation of hydrophobic active agents, substrates, or enzymes in aqueous environments. Dilute microemulsions have the viscosity of its continuous component (or the average of the both for the case of bicontinuous systems) [132] irrespective of their structure. An effective way to enhance the viscosity of droplet microemulsions is by the addition of telechelic polymers [133,134,135,136], which is interesting as for many applications of microemulsions an enhanced viscosity can be desirable. An example for the remarkable increase of viscosity that can be achieved by addition of telechelic, bridging polymers is shown in Figure 10. Here one sees an increase by about 4 orders of magnitude that takes place in a rather narrow range of polymer addition (where apparently the polymer concentration is sufficient to connect all the microemulsion droplets, which here is the case for having about 7-8 hydrophobic stearyl stickers per microemulsion droplet).

Another good reason for studying microemulsion networks is that they are good examples of model transient networks. The cross-linking points are the microemulsion droplets with typically very low polydispersity, that are located at a distance given by their number density and the telechelic polymer that connects them has a controlled length given by its molecular weight, persistence length and chain conformation. The stickers of the polymer solubilise into the microemulsion droplets uniformly [137]. If the drops are further apart than the chain length, the polymer forms loops with two ends localized in the same droplet. When the drops are closer than the end-to-end distance of the polymer, the polymer forms bridges between two droplets. The formation of bridges leads to the formation of clusters of droplets and, above the polymer percolation concentration, an infinite network of droplets spans the entire volume leading to a significant increase of the viscosity [136] (Figure 7C). 

Experimental results with small-angle neutron and X-ray scattering (SANS/SAXS) show that the structure of the microemulsion droplets in terms of shape and size is not affected by the addition of telechelic polymer. However, the polymer changes the interaction between the droplets [133,134,135,136]. The interaction has three contributions (see also Figure 8):

(1) The interaction between the droplets without polymer (excluded volume [135] or Yukawa repulsion for charged surfactants [138]);
(2) an entropic attraction induced by the bridging polymer [139];
(3) a soft repulsion caused by the self-excluding polymer chains between the droplets [92,140].

Depending on the relative importance of these contributions, the net interaction is attractive or repulsive [141]. 

The system exhibits a phase separation between a fluid sol phase and a polymer rich network phase when the net interaction is attractive enough [75]. The attractive interaction that leads to phase separation has a purely entropic origin since the increase in polymer configurations overcomes the entropy loss due to the phase separation and the formation of a dense phase [139]. Thus, it only depends on the relative length of the polymer compared to the separation between the droplets and not on the sticker length. It was experimentally demonstrated that the end-group does not influence the phase behaviour [75], but phase separation can be suppressed by introducing additional repulsive interactions [138]. 

Microemulsion networks exhibit viscoelastic behaviour with only one characteristic relaxation process described by a Maxwell model [134,142]. The relaxation time of the network is given by the residence time of the end group in the microemulsion droplet and, thus, depends on the length of the hydrophobic end-group [143]. 

The effect of the polymer architecture on the structure and dynamics of ME networks has also been studied [141,143]. Low functionality telechelic star polymers with 3 and 4 arms are able to interconnect microemulsion droplets. Neutron scattering experiments show that the attraction induced between the drops is larger with higher functionality polymers, which leads to a larger phase separation area in the polymer concentration-droplet concentration space. The repulsive component of the interaction potential, however only depends on the volume fraction of the hydrophilic chains. This leads to the observation that the local structure of the microemulsion networks is the same for the same polymer concentration, regardless of its functionality. Linear rheology experiments show that below the percolation threshold the viscosity is more influenced by the volume fraction of the created clusters. Above the percolation concentration higher polymer functionalities lead to a higher connectivity and, thus a higher elastic modulus.

In summary, it can be stated that the addition of hydrophobically modified polymers (especially telechelic ones) is a very attractive way of controlling the viscosity and gelation properties of otherwise low viscous microemulsions. The obtained rheological properties are controlled via the length of the hydrophobic sticker, the ratio of stickers per microemulsion droplets, the length of the hydrophilic chain, and the architecture of the bridging polymer.

### 5.3. Vesicles Interacting with Polymers

**Figure 11:**
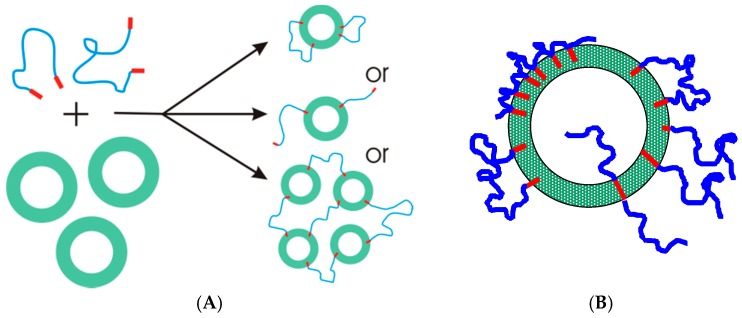
Schematic representation of the (**A**) association of telechelic polymers and vesicles leading to the formation of decorated vesicles and vesicle networks; and (**B**) anchoring of hydrophobically modified polymers of different architectures to vesicle membranes.

There is less work done on the fundamental properties on vesicles compared to microemulsions with added HM polymers. Such hydrophobically modified polymers can interact with vesicles and interconnect them within vesicle solutions [144,145,146,147,148] (Figure 11A). However, the vesicle/polymer systems are more complicated because, unlike in the case of microemulsions and micelles, the structure of the vesicles do not necessarily remain intact upon the addition of hydrophobically modified polymers. Anchored polymers in vesicle membranes (see Figure 11B) can have two effects: (1) change the curvature; (2) change the membrane gel to fluid transition temperature. In any case, the addition of hydrophobically modified linear polymers to vesicle solutions leads to the formation of networks. These networks have higher elasticity with higher polymer concentration [144,145,146]. Here again, it is necessary that the hydrophilic part of the polymer is longer than the distance between vesicle membranes to form bridges. 

Grafted polymers such as HM-chitosan also form vesicle networks [147,148]. This system is particularly advantageous because of its biocompatibility.

A very interesting case has been studied with catanionic vesicles composed of sodium dodecyl sulfate (SDS)/didodecyldimethylammonium bromide (DDAB) or sodium dodecylbenzenesulfonate (SDBS)/cetyltrimethylammonium tosylate (CTAT) in mixtures with hydrophobically modified sodium polyacrylate (hm-NaPA). When the vesicles are positively charged (as controlled by the SDS/DDAB or SDBS/CTAT ratio) they will form precipitates with the oppositely charged hm-NaPA but for the anionically charged vesicles one finds the formation of a gel already at as low concentrations of 1.25 wt % surfactant and 0.4 wt % hm-NaPA [149]. A similar behaviour has been reported for SDBS/CTAT vesicles when combined with hydrophobically modified (with *n*-dodecyl chains) chitosan, hm-chitosan, but interestingly no gelation was observed when adding the hm-chitosan to wormlike CTAT micelles. Here only an increase of viscosity and shift to a longer structural relaxation time takes place [147]. Gelation at higher polymer and surfactant concentration (but still in the 1 wt % regime) also has been reported for catanionic vesicles in the SDS/DDAB system upon admixing cationically modified cellulose (JR400 or LM200) [150]. Further studies then demonstrated that the hydrophobic modification present (a dodecyl chain employed for quaternization of the amine at the cellulose) in LM200 results in a more pronounced change of the rheological properties and the formation of more and more long-lived cross-links [144,145,146]. 

This principle of gelation was then also extended to phospholipids such as dipalmitoylphosphatidyl-choline (DPPC) and here similar gels with hm-chitosan can be formed which could be formulated as an injectable system for drug delivery as demonstrated with doxorubicin as model drug [151]. This type of vesicle/hm-chitosan gel then could also be transformed into a photoresponsive system by substituting the cationic surfactant by p-octyloxydiphenyl-iodonium hexafluoroantimonate (ODPI). ODPI can be regarded as a cationic surfactant but upon illumination with UV-light becomes decomposed into uncharged hydrophobic products, which leads to a transformation of the initially present vesicles into micelles, which macroscopically is seen as gel to sol transition [152]. 

## 6. Conclusions

This review discusses the structure and mechanical properties of gels formed by self-assembly of amphiphilic molecules at low and moderate concentration, i.e., well below dense packing of the molecules. There are several ways of achieving this. One way is by increasing the effective volume fraction via the enclosure of large volumes of solvent (the case of vesicles) or through the formation of supramolecular structures (like worm-like micelles) and network formation. The viscoelastic properties of these materials are given by the structure and the lifetime of the network bonds. In case of densely packed vesicles that time would be given by the cage opening of neighbouring vesicles. This time is typically much longer than the experimental window and thus effectively the system behaves as an elastic solid with a yield stress. The structural properties of worm-like micelle solutions are described by the micelle´s length, persistence length and concentration. Worm-like micelles have two relaxing mechanisms: the breaking and reforming of the micelles and the entanglement of cross-links, both determining the structural relaxation time τ_str_. In principle, worm-like micelles yield viscoelastic solutions, but once τ_str_ becomes sufficiently large one has effectively a gel as the flow becomes so slow that it is not observed within the experimental window. In the case of networks of associative polymers the number density of hydrophobic domains (polymeric or surfactant or microemulsions), the length and functionality of the polymer determine the structure, whereas the structural relaxation is mainly due to the residence time of the stickers in the hydrophobic domains (in the absence of entanglements). Especially the case of microemulsions is interesting as they are able to solubilise large amounts of active agents but for applications often a much enhanced viscosity is required as it can be obtained by the combination with amphiphilic polymers. 

Great advances in the synthesis of polymers and surfactants with controlled architecture and chemistry have been making the field moving fast towards systems of higher complexity and stimuli-responsiveness, where typically on has a response to changes of pH, temperature, or ionic strength. Mostly such systems work by interconnecting hydrophobic domains in aqueous solution, where the interaction is mostly of hydrophobic or electrostatic nature but also the chain entropy of the polymers may play a role. Stimuli-responsive systems are particularly interesting due to the possibility of switching from a liquid state to a gel state upon an external trigger, the same way nature does to create biological function. Especially here further future research advancements are to be expected. 

Therefore, in summary, it can be stated that it is possible to control the rheological properties of soft matter systems largely via the principles of self-assembly and this pertains also to the situation of forming practical gels, as they are often required for many practical formulations.
